# Effect of Diet on the Vitamin B Profile of Bovine Milk-Based Protein Ingredients

**DOI:** 10.3390/foods9050578

**Published:** 2020-05-04

**Authors:** Jonathan B. Magan, Tom F. O’Callaghan, Jiamin Zheng, Lun Zhang, Rupasri Mandal, Deirdre Hennessy, Mark A. Fenelon, David S. Wishart, Alan L. Kelly, Noel A. McCarthy

**Affiliations:** 1 Food Chemistry & Technology Department, Teagasc Food Research Centre, Moorepark, Fermoy, P61 C996 Cork, Ireland; Jonathan.Magan@teagasc.ie (J.B.M.); Tom.ocallaghan@teagasc.ie (T.F.O.); Mark.Fenelon@teagasc.ie (M.A.F.); 2 School of Food and Nutritional Sciences, University College Cork, T12 YT20 Cork, Ireland; a.kelly@ucc.ie; 3 The Metabolomics Innovation Centre, School of Biological Sciences, University of Alberta, Edmonton, AB T6G1C9, Canada; jiamin3@ualberta.ca (J.Z.); lun2@ualberta.ca (L.Z.); rmandal@ualberta.ca (R.M.); dwishart@ualberta.ca (D.S.W.); 4 Teagasc Animal and Grassland Research & Innovation Centre, Moorepark, Fermoy, P61 C996 Cork, Ireland; Deirdre.Hennessy@teagasc.ie

**Keywords:** Bovine diet, B vitamin composition, skim milk, sweet whey, acid whey, micellar casein whey

## Abstract

The influence of diet on the water-soluble vitamin composition of skim milk powder and whey protein ingredients produced from the milk of cows fed pasture or concentrate-based diets was examined. Fifty-one Holstein-Friesian cows were randomly assigned into three diets (*n* = 17) consisting of outdoor grazing of perennial ryegrass (GRS), perennial ryegrass/white clover (CLV), or indoor feeding of total mixed ration (TMR) for an entire lactation. Raw mid-lactation milk from each group was processed into skim milk powder and further processed to yield micellar casein whey and acid whey. Sweet whey was also produced by renneting of pasteurised whole milk from each system. The water-soluble vitamin profile of each sample was analysed using a combination of direct injection mass spectrometry and reverse-phase liquid chromatography–mass spectrometry. Vitamin B3 and B3-amide concentrations were significantly higher (*p* < 0.05) in TMR-derived samples than in those from CLV and GRS, respectively. Vitamin B1, B2, and B7 concentrations were significantly higher in GRS and CLV-derived samples than those from TMR. Significant differences in vitamins B1, B2, and B3-amide were also observed between protein ingredient types. This study indicates that bovine feeding systems have a significant effect on B vitamin composition across a range of protein ingredient types.

## 1. Introduction

Dairy protein commodities such as skim milk powder (SMP) and derivatives thereof (e.g., whey produced from milk acidification or membrane filtration) are widely used as the primary materials in dairy-based product formulations, particularly in the manufacture of infant milk formula, providing much of the protein, lactose and water-soluble micronutrients required for such formulations. Due to the high value of milk fat in comparison to skim milk, fat-filled milk powders can be produced at low cost by blending vegetable oils with SMP. However, with studies on the potential health benefits of milk fatty acids becoming increasingly positive [1,2], applications for milk fat in added-value nutritional beverages may become more widespread. This may contribute to reducing dependency on palm oil, the production of which is widely recognised as a major environmental issue [3]. 

Consumer awareness of sustainable food production is increasing [4,5], along with professional interest in communicating the concept of high nutrient-density foods to the consumer [6]. Milk and dairy products provide a good dietary source of water-soluble B vitamins, particularly vitamins B2 (riboflavin), B5 (pantothenic acid), and B12 (cobalamin), each with comparatively high bioavailability [7,8,9]. Milk is also a particularly good source of cobalamin as it is exclusively produced by soil bacteria and archaea [9], which can be consumed by grazing ruminants or by ruminal synthesis through the uptake of precursory cobalt from the soil [10]. Cobalamin cannot be acquired by humans from most dietary plant sources [11]. Previously, Jensen (1995) reported the standard quantities of water-soluble [12] and fat-soluble [13] vitamins in milk.

Consumer perceptions of healthier milk and dairy products increasingly tend towards pasture-based production systems [14]. Rising interest in pasture-derived milk is reflected by the increasing prevalence of dairy products marketed on the basis of claims of “pasture-fed” provenance. However, pasture-based milk production systems are climate-dependent and represent only a minor proportion of overall global milk production [15]. These systems are the most widely practiced in New Zealand and Ireland, where recent marketing efforts have been increasingly focused on the potential environmental [16] and health [17] benefits of their use. “Grass-fed” marketing claims have also become widespread among the protein supplement industry in the USA, where milk production is almost exclusively based on indoor, concentrate feeding-based systems [18]. Indeed, the vast majority of international milk suppliers use a concentrate-based system. For the consumer, concerns often emerge regarding animal welfare and the environmental impact of these systems [19]. These changing considerations have resulted in renewed interest in pasture-based milk production in the USA, where research into their economic merit is on-going. 

A significant effect of feeding system on the fatty acid profile of milk and dairy products has previously been shown [20,21]. While the effect of pasture or concentrate feeding on levels of fat-soluble vitamins such as retinol (vitamin A) and alpha-tocopherol (vitamin E) has been investigated in meat [22], neither the fat-soluble nor water-soluble vitamin profiles have been established for the milk of cows fed on these systems. Numerous studies [23,24,25] have investigated the effect of dietary supplementation of water-soluble vitamins (particularly biotin) on cow performance and health, though not on the subsequent vitamin composition in milk. Similarly, studies [26,27] have determined the effect of grass and concentrate feeding on water-soluble vitamin levels in rumen fluid and muscle tissue, but not in milk or milk-derived ingredients such as whey. Nonetheless, the importance of milk and dairy products as major sources of water soluble vitamins in human nutrition merits investigation of the variation in vitamin composition between milks derived from cows fed on different commonly practiced feeding systems.

Bovine milk is a source of water-soluble vitamins which are present in quantities that can contribute substantially to the minimum recommended daily allowance for children or adults. Skim milk powder also typically forms the nutritional base for infant milk formula (IMF) manufacture, where it is combined with lactose, demineralised whey, or whey protein concentrate to achieve an overall composition similar to that of human breast milk. Therefore, potential effects of ruminant feeding systems on the vitamin B content of skim milk and whey ingredients may be important considerations for formulation design and nutritional quality, particularly in relation to IMF production. 

With this considered, the objective of this study was to determine the influence of perennial ryegrass (*Lolium perenne* L.), perennial ryegrass/white clover (*Trifolium repens* L.), and indoor total mixed ration-based feeding systems on the water-soluble vitamin composition of SMP and whey protein ingredients.

## 2. Materials and Methods

### 2.1. Materials

Raw milk was obtained from Teagasc Animal and Grassland Research and Innovation Centre (Moorepark, Fermoy, Co. Cork, Ireland). Hydrochloric acid and sodium hydroxide used for acid whey production were sourced from Sigma Aldrich (Merck KGaA, Darmstadt, Germany). 

### 2.2. Experimental Design

This study utilised the same SMP and whey powders used by Magan et al. [28] and, as such, all experimental design conditions and powder production methods are as previously described. For detailed descriptions of the feeding system experimental design and the chemical composition of the feeds used in this study, see Egan et al. [29] and O’Callaghan et al. [30], respectively. Briefly, 54, 51, and 51 spring-calving Holstein-Friesian, with some Holstein-Friesian × Jersey cross-bred cows from the Teagasc Moorepark dairy herd were selected in 2015, 2016, and 2017, respectively, and randomly assigned to three groups (*n* = 18 in 2015; *n* = 17 in 2016 and 2017) with separate feeding systems. Cows were randomised by breed, calving date (mean 17 February; ±16 days), parity (2.45) and milk yield (23.7 kg) and milk solids (fat+protein) yield (2.04 kg) for the first 2 weeks post-calving. Mean body weight at the beginning of the experimental period was 519 kg. Group 1 was fed a total mixed ration (TMR) diet and housed indoors, Group 2 was maintained outdoors on perennial ryegrass only pasture (GRS), and Group 3 was also maintained outdoors on a perennial ryegrass/white clover pasture (CLV) with an average annual sward white clover content of 24%. On a dry matter (DM) basis, the TMR diet consisted of 8.3 kg of concentrates, 7.15 kg of grass silage, and 7.15 kg of maize silage. Individual electronically controlled Griffith Elder Mealmaster feed bins (Griffith Elder and Company Ltd., Suffolk, England) were used to administer adlibitum feed to the cows within the TMR system at 08:30 h daily. Both groups of pasture based cows consumed ~18 kg DM/day. This was allocated using estimates of pre-grazing herbage mass and daily post grazing sward heights as described by Egan et al. [29]. Each group of cows was milked twice daily at 07:30 and 15:30 h. Cows from each of the three feeding systems were milked separately and their milk was segregated into designated 5000 L refrigerated tanks. Tanks were maintained at 4 °C with morning and evening milk being added together and agitated prior to sample collection. 

The raw milk of each of the three groups of 17 cows was bulked into three designated bulk tanks over 5 consecutive milkings (3 morning milkings, 2 evening milkings) and collected on two separate occasions over a two-week period in July 2017 (mean days in milk 143) and processed into two batches of skim milk powder (SMP) from each feeding system at Moorepark Technology Ltd. (Moorepark, Fermoy, Co. Cork, Ireland). All of the milk powders within each batch were manufactured on the same day.

### 2.3. Protein Ingredient Manufacture

Each SMP, sweet whey, micellar casein whey, and acid whey powder was produced as described in detail in Magan et al. [28]. Briefly, low-heat non-agglomerated SMP was produced from pasteurised, separated, and spray-dried raw whole milk from each group of cows at pilot plant scale. Sweet whey powder was produced at laboratory scale by the addition of chymosin (Chy-Max Plus, 200 IMCU mL^−1^; Chr Hansen Ireland Ltd., Cork, Ireland) to pasteurised whole milk and subsequent curd cutting and whey drainage. Acid whey was produced at laboratory scale by acidification of reconstituted SMP from each feeding system, followed by curd cutting and whey drainage. Both sweet whey and acid whey were filtered using Whatman No. 1 filter paper and clarified using a 0.1-μm Sartocon Slice polyethersulfone cassette membrane (Sartorius AG, Göttigen, Germany). Micellar casein whey was produced at laboratory scale by filtering reconstituted SMP from each feeding system using this membrane. Each whey type was then freeze-dried in a Labconco stoppering tray dryer equipped with a Freezone 12 plus vacuum collector/refrigerator unit (Labconco, Kansas City, MO, USA) to yield whey powder. 

### 2.4. Liquid Chromatography–Mass Spectrometry (LC-MS/MS)

#### 2.4.1. Sample Preparation

Water-soluble vitamin (B1, B2, B3, B3-amide, B5, B6-Pyridoxine, and B7) analysis was carried out at The Metabolomics Innovation Centre (University of Alberta, Edmonton, Alberta, Canada) with a targeted mass spectrometry (multiple reaction monitoring) method using a reverse-phase LC-MS/MS assay. Standard solutions, internal standard solution, and quality control solutions were all diluted using 0.1% formic acid in deionised water. Internal standard solution (10 μL) was first added to 0.6 mL Eppendorf tubes. Calibration standard solutions (50 μL), quality control standard solutions (50 μL), and reconstituted skim milk/whey samples (50 μL) were then added to their corresponding Eppendorf tubes. Protein was precipitated by the addition of 60 μL of trichloroacetic acid (50 mg mL^−1^) to each tube, after which each sample was vortexed for 30 s. All tubes were stored on ice for 4 h, followed by centrifugation at 13,000 rpm for 15 min. The supernatant from each tube was then transferred into a 96 deep-well collection plate and sealed with a pre-slit mat. 

#### 2.4.2. Operating Conditions

The aqueous phase (solvent A) consisted of 5 mM ammonium formate and 0.1% formic acid in water, while the organic phase (solvent B) consisted of 5 mM ammonium formate and 0.1% formic acid in methanol. Samples were separated using an Agilent reversed-phase Zorbax Eclipse XDB C18 (3.0 mm × 100 mm, 3.5 μm particle size, 80 Å pore size) column (Agilent Technologies, Santa Clara, CA, USA) at a column temperature of 40 °C. Samples were injected at a volume of 10 μL at an auto-sampler temperature of 4 °C. Flow rate was 400 μL min^−1^ and run time was 9.5 min. Mass spectrometric analysis was performed on an AB SciexQTrap^®^ 4000 tandem mass spectrometry instrument (Applied Biosystems/MDS Analytical Technologies, Foster City, CA, USA). Multiple reaction monitoring was carried out in positive ionisation mode at a temperature of 450 °C and an ion spray voltage of 5500 V. Curtain gas, gas stream 1, and gas stream 2 pressures were 20, 40, and 60 psi, respectively. All 48 samples were analysed together in the same run (24 samples in duplicate). Validation parameters (calibration range, calibration regression coefficient, concentrations (µM) of quality control solutions, % accuracy, precision and recovery, limit of detection, and limit of quantitation) for each vitamin in the assay are shown in Supplementary Materials Table S1. Quality control samples were run in triplicate to calculate average accuracy (80%–120%) and precision (within 20%) percentages. Unspiked samples, together with low, medium, and high-spiked samples were measured in triplicate to calculate recovery percentages (80%–120%). Data analysis and calculations of vitamin concentrations were carried out using Analyst Software 1.6.2. 

### 2.5. Nephelometry

The vitamin B12 content of each SMP sample was determined by nephelometry by an external laboratory (Eurofins Food Testing Ireland—EFTI Cork, Glanmire Industrial Estate, Glanmire, Co. Cork) using the AOAC 952.20 microbiological assay method [31]. Briefly, vitamin B12 was extracted from the sample in an autoclave using a buffered solution. After dilution with basal medium (containing all required growth nutrients except cobalamin), the growth response of *Lactobacillus leichmanii* (ATCC 7830) to extracted cobalamin was measured turbidimetrically and compared to calibration solutions of known concentrations. Vitamin B12 concentrations were measured only in the SMP samples.

### 2.6. Statistical Analysis

Statistical analysis was performed using Genstat v18.1 (VSN International Ltd., Hemel Hempstead, Hertfordshire, UK). The mean of two replicates was used for each sample value. Datasets were analysed for normality using the Shapiro–Wilk’s test. Data was deemed normally distributed and analysis was carried out using a 4 × 3 factorial ANOVA with post hoc Tukey test. The effect of ingredient type (4 levels: SMP, sweet whey, MCW, and acid whey) and the effect of cow diet (3 levels: GRS, CLV, and TMR) were considered in the ANOVA. *p*-values < 0.05 were considered significant. Multivariate analysis of the vitamin profiles was also performed. A supervised multivariate model was built using partial-least-square discriminant analysis (PLS-DA). To validate the model, a permutation test with 2000 repetitions was performed to check that the model differed from a random model (*p* < 0.05). In addition, the *R*^2^ and *Q*^2^ parameters were obtained to assess the performance of the model using 10-fold cross validation approach. The variables which have the greater influence on the latent variables of the built model were determined using a variable importance plot (VIP). Unsupervised hierarchical clustering analysis (HCA) was performed to observe patterns in the data, and is shown as a heatmap. Each of these tests and generation of subsequent figures were carried out using Metaboanalyst 3.0 software (www.metaboanalyst.ca) [32].

## 3. Results and Discussion

### 3.1. Overall Distribution

**Figure 1:**
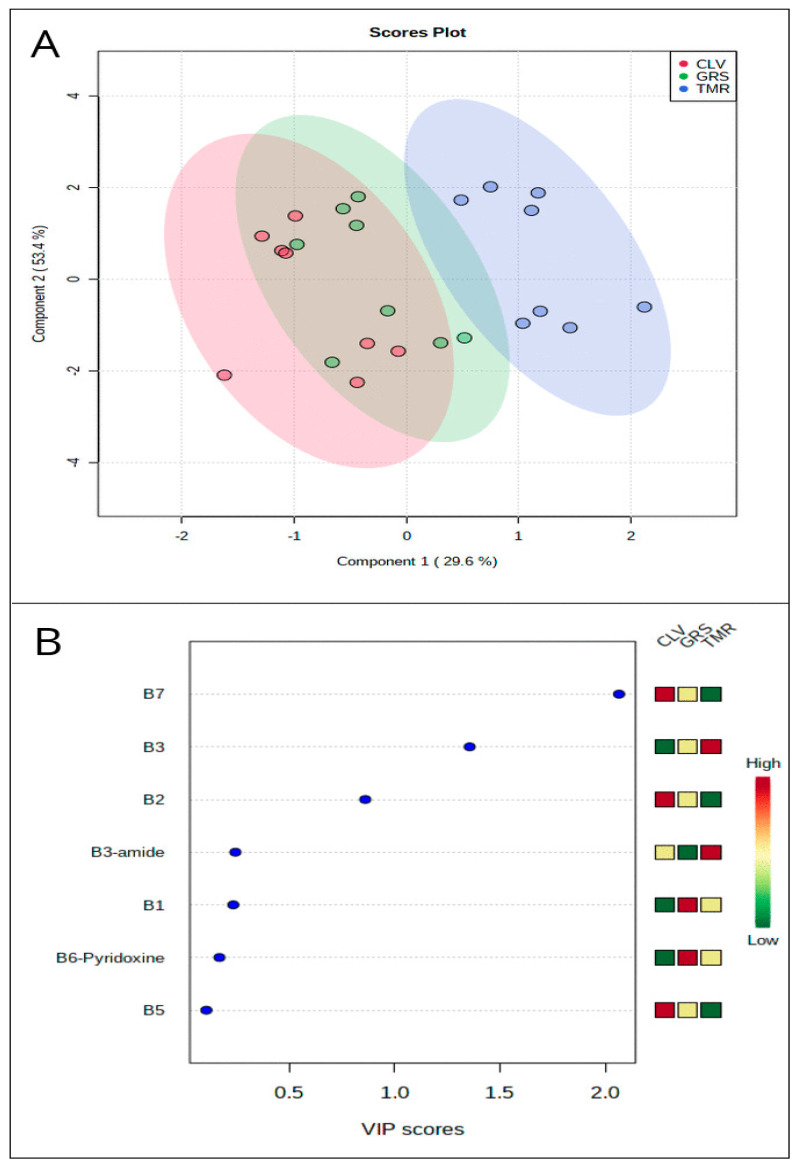
Panel (**A**): partial-least-square discriminant analysis (PLS-DA) score plot for water-soluble vitamins in skim milk powder and whey ingredients from perennial ryegrass (GRS), perennial ryegrass/white clover (CLV), and total mixed ration (TMR) feeding systems, determined by LC-MS/MS (*R*^2^ = 0.80, *Q*^2^ = 0.71). Panel (**B**): variable importance plot of vitamins most responsible for separation observed in PLS-DA.

**Figure 2:**
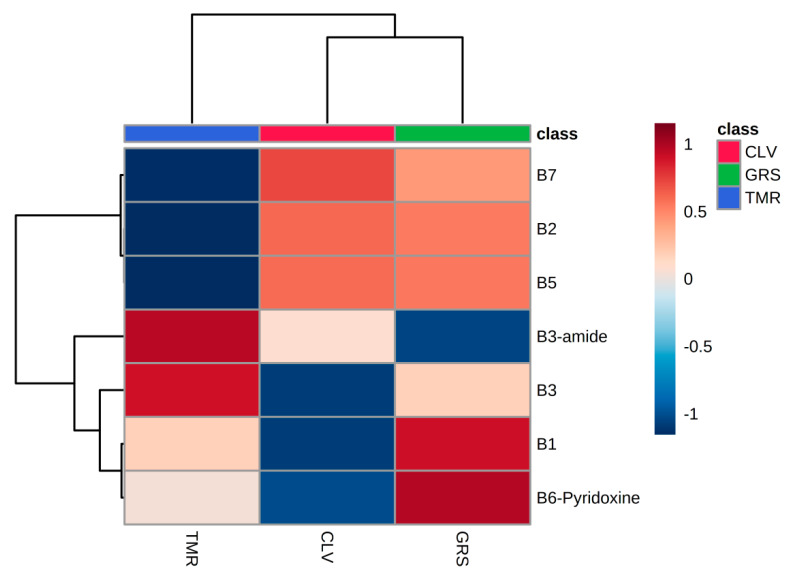
Hierarchical clustering analysis of average skim milk powder and whey ingredient vitamins from perennial ryegrass (GRS), perennial ryegrass/white clover (CLV), or total mixed ration (TMR) feeding systems, determined by LC-MS/MS. Degree of positive and negative correlation between vitamin and diet is indicated by +1 (red) to −1 (blue).

Table 1 shows the average concentration of each water-soluble vitamin expressed as μg per g of protein in each ingredient type. Average concentrations (µM) for each vitamin in SMP at 9.5% total solids and each whey type at 6.5% total solids from each feeding system are shown in Supplementary Materials Table S2. Table 2 compares the average concentration of each water-soluble vitamin in the sweet whey, micellar casein whey (MCW), and acid whey samples, expressed as μg per g of protein. Average concentrations (µM) for each vitamin in each whey type at 6.5% total solids are shown in Supplementary Materials Table S3. Table S4 in Supplementary Materials shows average concentrations (μg/g protein) for each vitamin in sweet whey, micellar casein whey, and acid whey powders derived from each feeding system. The overall significant effect of feeding system on vitamin profile can be shown using partial-least-square discriminant analysis (PLS-DA) (Figure 1, panel A) and hierarchical clustering analysis (Figure 2). Figure 1A shows a substantial overlap in the distribution of GRS and CLV samples and a more pronounced separation between both and the TMR samples. In contrast to the clustered variables of the GRS and CLV systems, each TMR variable was distinct from those of the other two systems. In this PLS-DA, *R*^2^ = 0.80 and *Q*^2^ = 0.71, indicating a close fit to predicted variation, with 83% of the observed variance also being explained by the model. Figure 1B shows the variable importance plot (VIP) generated from this PLS-DA, which determines the variables which contribute most to the observed variance in the model and indicates that vitamins B7, B3, and B2 contribute most to the discrimination between classes. The degree of positive or negative correlation of each vitamin to a particular feeding system is shown in Figure 2, which shows the notably positive correlation between the level of vitamin B3 complex and the TMR feeding system and the respective negative correlations of nicotinamide with the GRS system and nicotinic acid to the CLV system. The positive correlation of riboflavin and biotin to both the GRS and CLV systems is also apparent from Figure 2, while these vitamins exhibit a strongly negative correlation with the TMR system. The relative abundance of particular vitamins in milk from different feeding systems may thus be used as an effective means of differentiation between products derived from these different systems. The potential to distinguish between pasture-derived and concentrate-derived milks based on the metabolomics profile has also previously been determined in raw milk [33], SMP, and whey ingredients [28] using quantitative nuclear magnetic resonance (1H-NMR) and reverse-phase liquid chromatography–mass spectrometry (LC-MS/MS), respectively. 

### 3.2. Vitamin Composition

#### 3.2.1. Vitamin B1 (Thiamine)

Concentrations of thiamine in GRS and CLV-derived SMP samples were significantly higher (*p* < 0.05) than those derived from TMR (Table 1), despite higher concentrations of thiamine typically being present in the germ and seed of cereal grains than other plant sources [34]. Differences between diet were not observed in any of the whey samples, however, Pan et al. [35] suggested that ruminal thiamine production may be reduced in low rumen pH caused by subacute ruminal acidosis arising from feeding of high levels of concentrates. A previous study by Duckett et al. [36] also found thiamine concentrations to be three times greater in the muscle tissue of grass-fed bulls compared to those fed on a high-concentrate diet, while Shingfield et al. [37] found no significant variation in the thiamine content of milk derived from cows fed varying levels of concentrates. Thiamine is, however, stored in particularly high concentrations in animal muscle tissue [38]. Thiamine content was also shown to be significantly different between whey ingredient types in the present study (Table 2). Average concentrations of thiamine in sweet whey (31.2 µg/g protein) were significantly higher (*p* < 0.05) than for both MCW (22.3 µg/g protein) and acid whey (27.3 µg/g protein). Thiamine content is associated with protein content, as serum thiamine is primarily bound to albumin [39]. The SMP and whey powders used in this study are the same as those previously used by Magan et al. [28], where the total protein content of the powders was determined, with sweet whey exhibiting higher average total protein content (9.44%) than MCW (7.76%) and acid whey (7.71%). 

#### 3.2.2. Vitamin B2 (Riboflavin)

Average concentrations of riboflavin were significantly (*p* < 0.05) higher in both GRS and CLV samples when compared to the TMR sample (Table 1). Bovine dietary riboflavin is primarily sourced from green, leafy forage, though riboflavin synthesis also occurs in the rumen [40]. Riboflavin provides pigmentation in leaves [41], conferring a yellow colour similar to β-carotene, the relative abundance of which is primarily responsible for the intensity of yellow colour in fat-containing dairy systems [30,42]. Although each of the ingredient types in the present study are derived from non-fat systems and β-carotene is fat-soluble, visible differences in yellowness were observed between samples derived from each feeding system. Riboflavin is present in considerably lower concentrations in cereal grains, compared to fresh leafy forage (i.e., grass) [43]. 

Previous studies by Hayes et al. [44] and Duckett et al. [36] reported increased riboflavin concentrations in the rumen fluid of bulls receiving increased dietary roughage (i.e., hay) content and in the muscle tissue of bulls assigned to a pasture-based, rather than high concentrate-based finishing system, respectively. Conflicting results were found in a study by Santschi et al. [26], where increased riboflavin content was recorded in the rumen fluid of cows fed at a high concentrate to forage ratio. Poulsen et al. [45] compared the riboflavin content of bulk milk from three dairies in Denmark, recording higher riboflavin concentrations in milk from an organic dairy derived from high dietary proportions of grass and legume-based forage, when compared to the milk from two conventional dairies. The average riboflavin concentration of sweet whey (1169 µg/g protein) was significantly higher (*p* < 0.05) than both MCW (166 µg/g protein) and acid whey (71.9 µg/g protein) (Table 2). Previously, Mavropoulou and Kosikowski [46] examined commercially produced spray dried whey samples and found higher concentrations of riboflavin in sweet whey than in acid whey powders (data presented on a g per kg powder basis). Glass and Hedrick [47] reported similarly increased riboflavin content in commercial dried sweet whey compared to acid whey (data presented on a mg per 100 g powder basis). However, the differences observed in both of the previous studies are significantly lower than those for the present study. The exact reason for the present finding is still not fully understood.

#### 3.2.3. Vitamin B3 (Nicotinic Acid)

The vitamin B3 complex comprises two common forms; nicotinic acid, nicotinamide, and a third recently discovered form; nicotinamide riboside [48]. Both nicotinic acid and nicotinamide are identified in the LC-MS/MS analysis used in the present study, though nicotinamide is present in substantially higher concentrations in bovine milk than nicotinic acid as the latter is converted to the amide form in the rumen [49]. The average concentration of nicotinic acid in ingredients derived from TMR was significantly higher (*p* < 0.05) than those from CLV, while the nicotinamide concentration of TMR-derived ingredients was significantly higher than those derived from GRS (Table 1). The bioavailability of both forms of vitamin B3 is equivalent [50].

Primary bovine dietary sources of nicotinic acid are cereal grains, although, similar to riboflavin, synthesis of nicotinic acid also occurs in the rumen [51]. The high inclusion rate of cereal-derived concentrates in the TMR feeding system is likely the most significant contributor to the increased nicotinic acid content in these samples. Hayes et al. [41] showed increased levels of nicotinic acid in the rumen fluid of bulls fed a concentrate-based diet. Nicotinic acid is synthesised from the essential amino acid tryptophan [52], though previous analysis of the tryptophan content of the samples used in the present study did not reveal significant differences between the diets [28]. The lack of significant variation in tryptophan content between the diets relative to nicotinic acid content may be explained by the primary utilisation of tryptophan for protein synthesis [53] and the relative inefficiency of the synthesis of nicotinic acid from tryptophan [54]. Contrary to riboflavin, average nicotinamide concentrations of MCW (134 µg/g protein) and acid whey (138 µg/g protein) were significantly higher than those of sweet whey (79.6 µg/g protein) (Table 2). 

#### 3.2.4. Vitamin B5 (Pantothenic Acid) and B6 (Pyridoxine)

While concentrations of pantothenic acid were high in all samples, particularly when compared to the low concentrations of pyridoxine (Table 1), both vitamin B5 and pyridoxine were not found to be significantly different between feeding systems or ingredient types. However, the forage:grain ratio of foodstuffs consumed by the cow is generally regarded as an influence on pantothenic acid synthesis [55]. The effect of varying this ratio on the levels of pantothenic acid transferred into the milk of the cow has not previously been investigated. Sources of vitamins B5 and B6 are consistent in the bovine diet, with relatively similar concentrations present in leafy forages and cereal grains [55,56]. A study comparing the vitamin B6 content of commercial milk with that of milk produced from cows fed a diet restricted in B6 found consistent levels of the vitamin between both milk sources, indicating that the concentrations of vitamin B6 present in milk may be independent of diet and it may instead be entirely supplied through ruminal synthesis [57]. 

The vitamin B6 complex consists of six vitamers: pyridoxine, pyridoxal, pyridoxamine, and the phosphate ester form of each; pyridoxine phosphate, pyridoxal phosphate, and pyridoxamine phosphate [58]. The multiple reaction monitoring assay carried out in the present study exclusively measured the pyridoxine vitamer, while the predominant vitamer present in bovine milk is the active form pyridoxal phosphate [59]. Consequently, concentrations for vitamin B6 observed in this study were negligible in comparison to average values for total vitamin B6 observed for milk in the literature [12,60,61]. 

#### 3.2.5. Vitamin B7 (Biotin)

The average biotin concentrations of CLV and GRS were significantly higher (*p* < 0.05) than that of TMR (Table 1). Biotin is present in low concentrations in milk [62], though milk remains a good dietary source as requirements for the vitamin are comparatively low [63]. As with the other B-complex vitamins, biotin is a product of rumen metabolism [24], though conflicting information exists on the relationship between dietary intake, net ruminal synthesis, and duodenal flow of biotin [64,65,66]. However, the analysis carried out herein suggests that biotin content was significantly affected by feeding system. 

The role of nutrition in biotin synthesis has been widely investigated. Briggs et al. [67] suggested that feeding of dietary urea may increase rumen biotin synthesis, which corresponds to the increased concentration of the vitamin in the CLV sample in the present study. Increased urea content has previously been observed in samples derived from this system [33], while increasing dietary biotin supplementation was found to significantly increase milk biotin concentrations [24]. Other studies have outlined a decrease in rumen biotin content with increasing grain content [68] or decreasing dietary forage content [26] and an increase in rumen acidity with increasing grain supplementation, leading to inhibition of cellulolytic rumen microflora [69], as previously suggested for thiamine content in Section 3.2.1. This is supported by O’Callaghan et al. [33], who showed no significant effect of bovine feeding system on the overall composition of rumen microflora, but rather rumen microflora functionality. The results of the present study support this indirect effect of the feeding system, whereby the functionality of rumen microflora and, hence, efficiency of rumen biotin synthesis may be increased or decreased depending on the substrate derived from the type of feed consumed by the cow. However, this is a notable contrast to pantothenic acid and pyridoxine (Section 3.2.4), which are primarily products of rumen metabolism, but do not display a significant effect of the feeding system on their concentrations in the SMP and whey samples.

#### 3.2.6. Vitamin B12 (Cobalamin)

Cobalamin concentrations did not differ significantly (*p* > 0.05) between SMP samples. The highest concentrations were present in CLV-derived SMP (38.6 μg kg^−1^), followed by TMR (37.2 μg kg^−1^) and GRS (34.3 μg kg^−1^). This is likely due to increased levels of cobalt present in the concentrate used in the TMR ration and in the root nodules of the CLV sward. Leguminous plants such as *Trifolium repens* L. exhibit a high affinity for the concentration of cobalt [70], which is required by nitrogen-fixing microflora present in the root nodule [71] and forms the central constituent in cobalamin synthesis [72]. The lower concentrations observed in GRS-derived SMP may therefore be due to the absence of cobalt from the leaves of the perennial ryegrass exclusively consumed by the cows assigned to this feeding system.

### 3.3. Relationship between Skim Milk and Recommended Daily Allowances

Table 3 shows the recommended daily allowance (RDA) and adequate intake (AI) values for each water-soluble vitamin for mature females and males (aged 14+) [63], along with the mass of each vitamin present in a 200-mL serving of skim milk derived from each feeding system and the percentage which they contribute to the RDA for each vitamin. Skim milk from each diet offers a low proportion of the RDAs for vitamins B1 and B3, though the stated allowance for vitamin B3 represents “niacin equivalent”, a figure which incorporates the typical daily intake of tryptophan, the mass of which is not included in the skim milk sample values in the table. As discussed in Section 3.2.4, the proportion of vitamin B6 in each SMP refers to the pyridoxine form only and is, therefore, omitted from Table 3, as an approximate comparison to the full RDA for vitamin B6 cannot be made for the individual vitamer. The vitamin B5 content of skim milk from each feeding system would account for 16% to 17% of its RDA, while, notably, almost one third of the average RDA (RDA based on recommendations of US Institute of Medicine [63]) for vitamin B12 would be provided from a 200-mL serving of skim milk from each feeding system (Table 3). As previously described, concentrations of vitamins B2 and B7 were approximately twice as high in GRS and CLV-derived samples with respect to TMR-derived samples. Thus, approximately 10% of the RDA of vitamin B7 may be provided by a skim milk serving from either pasture-derived sample, with approximately 5% available from TMR-derived milk. While this ratio is similar for vitamin B2, the riboflavin content of a 200-mL serving of skim milk from each feeding system would provide substantially higher than the average RDA for the vitamin.

### 3.4. Vitamin Content of Skim Milk for Use in Infant Milk Formula Manufacture

Table 4 shows the B vitamin contribution of SMP in an IMF (1.4%, *w/w*, protein), if 50% (*w/w*) of the total protein is obtained from SMP. In addition, shown are the minimum amount of each vitamin required (mg/100 mL), based on the recommendations of the Codex Alimentarius standard 72 [73] for IMF with an energy density of 65 kcal/100 mL. While further quantities of water-soluble vitamins would be introduced to an IMF mixture through the addition of whey protein ingredients or by supplementation, the SMP base from any of the three feeding systems would provide substantially more riboflavin than the minimum required level. Similarly, the required cobalamin content would be achieved using CLV (0.000068 mg/100 mL) or TMR-derived SMP (0.000065 mg/100 mL) alone, with GRS-derived SMP only slightly lower at 0.000060 mg/100 mL. The use of SMP derived from GRS, CLV, and TMR would provide 30%, 35%, and 16% of the required biotin content, respectively, while each SMP type would provide approximately 30% of the pantothenic acid requirement. Quantitative differences in thiamine, nicotinic acid, and pyridoxine content between the three feeding systems are unsubstantial when compared to the high requirements for these vitamins. This necessitates the use of vitamin concentrate pre-mixes to supplement the required levels for IMF. As discussed in Section 3.2.4, the predominant form of vitamin B6 in bovine milk is pyridoxal phosphate, whereas in human milk, pyridoxal is the dominant form, followed by pyridoxal phosphate, with the other vitamers present in very low concentrations [58]. Fortification of most foods, including IMF, is most commonly achieved through the addition of pyridoxine alone in the form of the salt pyridoxine hydrochloride [74]. 

## 4. Conclusions

The utilisation of pasture or concentrate-based bovine feeding systems significantly affected the relative concentrations of a limited number of water-soluble vitamins in the skim milk and whey protein powder ingredients used in this study and varied across ingredient types. Significant differences in thiamine (B1) content were observed only in SMP, while significantly higher riboflavin (B2) content was apparent in GRS and CLV-derived SMP, sweet whey, and MCW, when compared to TMR. This may be primarily attributable to the forage content of the GRS and CLV diets which contain high concentrations of riboflavin. Despite the higher proportions of the nicotinic acid/amide complex in cereal grains, significantly higher concentrations of vitamin B3 and B3-amide were observed only in TMR-derived sweet whey and acid whey, respectively. Variations in the concentration of biotin (B7) may, however, be indirectly affected by bovine diet, through substrate-based modulation of rumen microflora and subsequent variation in the relative efficiency of rumen biotin synthesis. The PLS-DA, VIP, and HCA plots provide a visual representation of the distinguishable difference in milk protein ingredients derived from each feeding system based on their vitamin profile, which offers potential as a means of milk product verification. Riboflavin and biotin exhibited the most biologically significant differences in SMP when compared to average recommended daily allowances and requirements for infant milk formulation. The data presented also suggest that significant differences in vitamin content may arise due to the type of whey production method used, independent of dietary effects.

## Figures and Tables

**Table 1:** Average concentrations (μg/g protein) of water-soluble vitamins for skim milk, sweet whey, micellar casein whey, and acid whey powders derived from the milk of Holstein-Friesian cows assigned to perennial ryegrass (GRS), perennial ryegrass/white clover (CLV), and total mixed ration (TMR) feeding systems, determined by LC-MS/MS.

Sample Type	Water-Soluble Vitamin (μg/g Protein)	GRS	CLV	TMR
Skim milk powder	B1	5.47 ^b^	5.44 ^b^	4.31 ^a^
	B2	422 ^b^	432 ^b^	250 ^a^
	B3	0.86	0.63	0.94
	B3-amide	15.4	15.4	18.0
	B5	112	117	125
	B6-Pyridoxine	0.13	0.11	0.13
	B7	0.46	0.53	0.25
Sweet whey powder	B1	29.5	27.9	36.3
	B2	1489 ^b^	1400 ^b^	636 ^a^
	B3	4.60 ^a^	3.37 ^a^	5.41 ^b^
	B3-amide	74.9	82.0	81.9
	B5	738	695	675
	B6-Pyridoxine	0.71	0.77	0.77
	B7	3.04 ^a,b^	3.12 ^b^	1.23 ^a^
Micellar casein whey powder	B1	23.2	22.1	20.9
	B2	191 ^b^	232 ^b^	85.2 ^a^
	B3	4.48	4.51	4.91
	B3-amide	117	150	137
	B5	869	1067	811
	B6-Pyridoxine	0.77	0.86	0.74
	B7	3.97 ^b^	5.65 ^b^	1.77 ^a^
Acid whey powder	B1	30.9	22.4	29.4
	B2	81.7	83.8	49.6
	B3	5.89	4.03	8.03
	B3-amide	110 ^a^	115 ^a,b^	129 ^b^
	B5	929	843	933
	B6-Pyridoxine	1.03	0.77	0.80
	B7	3.55 ^b^	3.75 ^b^	1.95 ^a^

Values are presented as the average of duplicate samples. GRS—Cows fed perennial ryegrass only. CLV—Cows fed perennial ryegrass/white clover. TMR—Cows fed total mixed ration adlibitum. Vitamins: B1—Thiamine, B2—Riboflavin, B3—Nicotinic acid, B3-amide—Nicotinamide, B5—Pantothenic acid, B7—Biotin. Note: Only the pyridoxine form of vitamin B6 is represented in the data. ^a,b^ Different superscripts within a row indicate significant differences (*p* < 0.05).

**Table 2:** Average concentrations (μg/ g protein) of total water-soluble vitamins for sweet whey, micellar casein whey, and acid whey powders derived from the milk of Holstein-Friesian cows assigned to each feeding system, determined by LC-MS/MS.

Water-Soluble Vitamin (μg/g Protein)	Sweet Whey Powder	Micellar Casein Whey Powder	Acid Whey Powder
B1	31.2 ^b^	22.1 ^a^	27.6 ^a^
B2	1175 ^b^	170 ^a^	71.7 ^a^
B3	4.46	4.64	5.98
B3-amide	79.6 ^a^	135 ^b^	118 ^b^
B5	703	916	902
B6-Pyridoxine	0.75	0.79	0.87
B7	2.46	3.80	3.08

Values are presented as the average of data from duplicate samples. Vitamins: B1—Thiamine, B2—Riboflavin, B3—Nicotinic acid, B3-amide—Nicotinamide, B5—Pantothenic acid, B7—Biotin. Note: Only the pyridoxine form of vitamin B6 is represented in the data. ^a,b^ Different superscripts within a row indicate significant differences (*p* < 0.05).

**Table 3:** Recommended daily allowances (US Institute of Medicine, 1998) for females and males (aged 14+) and average mass (mg) of water-soluble vitamins present in skim milk powder reconstituted at 9.5% total solids derived from Holstein-Friesian cows assigned to each feeding system.

Water- Soluble Vitamin	Recommended Daily Allowance (mg)		Mass (mg) in 200 mL Skim Milk	% of Adult Human RDA		Mass (mg) in 200 mL Skim Milk	% of Adult Human RDA		Mass (mg) in 200 mL Skim Milk	% of Adult Human RDA	
	Female	Male	GRS	Female	Male	CLV	Female	Male	TMR	Female	Male
B1	1.1	1.2	0.038	3.5	3.2	0.038	3.5	3.2	0.029	2.6	2.4
B2	1.1	1.3	2.964	269	228	3.049	277	235	1.697	154	131
B3 complex	14	16	0.114	0.8	0.7	0.113	0.8	0.7	0.128	0.9	0.8
B5	5.0 *	5.0 *	0.789	16	16	0.829	17	17	0.846	17	17
B7	0.03 *	0.03 *	0.0033	11	11	0.0037	12	12	0.0017	5.7	5.7
B12	0.0024	0.0024	0.0006	27	27	0.0007	30	30	0.0007	29	29

Skim milk values are presented as the average of duplicate samples. GRS—Cows fed perennial ryegrass only. CLV—Cows fed perennial ryegrass/white clover. TMR—Cows fed total mixed ration adlibitum. Vitamins: B1—Thiamine, B2—Riboflavin, B3 complex—Nicotinic acid and Nicotinamide, B5—Pantothenic acid, B7—Biotin, B12—Cobalamin. * Indicates adequate intake values where recommended daily allowance values have not been derived. Note: Recommended daily allowance values for the vitamin B3 complex are expressed as “niacin equivalent”, comprising vitamin B3, B3-amide, and tryptophan. Values for the vitamin B3 complex are expressed as the sum of vitamin B3 and B3-amide.

**Table 4:** B-vitamin contribution (mg) of skim milk powder derived from each feeding system in infant milk formula (65 kcal per 100 mL).

Water-Soluble Vitamin	Mass (mg/100 mL)			
	GRS	CLV	TMR	Minimum Requirement (CODEX STAN 72, 1981)
B1	0.004	0.004	0.003	0.039
B2	0.279	0.287	0.160	0.052
B3 complex	0.011	0.011	0.012	0.195
B5	0.074	0.078	0.080	0.260
B6-Pyridoxine	0.000088	0.000075	0.000086	0.023
B7	0.0003	0.0003	0.0002	0.001
B12	0.000060	0.000068	0.000065	0.000065

SMP values are presented as the average of duplicate samples. GRS—Cows fed perennial ryegrass only. CLV—Cows fed perennial ryegrass / white clover. TMR—Cows fed total mixed ration adlibitum. Vitamins: B1—Thiamine, B2—Riboflavin, B3 complex—Nicotinic acid and Nicotinamide, B5—Pantothenic acid, B7—Biotin, B12—Cobalamin. Note: Only the pyridoxine form of vitamin B6 is represented in the data. Values for the vitamin B3 complex are expressed as the sum of vitamin B3 and B3-amide.

## Supplementary Materials

The following are available online at https://www.mdpi.com/2304-8158/9/5/578/s1, Table S1: Validation parameters for water-soluble vitamin determination by LC-MS/MS, Table S2: Average concentrations (μM) of water-soluble vitamins for reconstituted skim milk (9.5% total solids), sweet whey (6.5% total solids), micellar casein whey (6.5% total solids) and acid whey(6.5% total solids) powders derived from the milk of Holstein-Friesian cows assigned to perennial ryegrass (GRS), perennial ryegrass/white clover (CLV) and total mixed ration (TMR) feeding systems, determined by LC-MS/MS, Table S3: Average concentrations (μM) of total water-soluble vitamins for reconstituted rennet whey (6.5% total solids), micellar casein whey (6.5% total solids) and acid whey (6.5% total solids) powders derived from the milk of Holstein-Friesian cows assigned to each feeding system, determined by LC-MS/MS. Table S4: Average concentrations (μg/g protein) of water-soluble vitamins for sweet whey, micellar casein whey and acid whey powders derived from the milk of Holstein-Friesian cows assigned to from perennial ryegrass (GRS), perennial ryegrass/white clover (CLV) and total mixed ration (TMR) feeding systems, determined by LC-MS/MS.
