# Supplementary material for: Effect of Diet on the Vitamin B Profile of Bovine Milk-Based Protein Ingredients
Source: Foods. 2020 May 4;9(5):578. doi: 10.3390/foods9050578 (PMC7278826; doi:10.3390/foods9050578)
Supplement: Supplementary file 1 [file foods-09-00578-s001.pdf]

Supplementary data

Table S1: Validation parameters for water-soluble vitamin determination by LC-MS/MS.

		**Vitamin B1**	**Vitamin B2**	**Vitamin B3**	**Vitamin B3-amide**	**Vitamin B5**	**Vitamin B6-Pyridoxine**	**Vitamin B7**
Calibration Ranges (μM)		0.01 - 1	0.01 - 1	0.1 - 10	0.05 - 5	0.05 - 5	0.01 - 1	0.01 - 1
Calibration Regression R^2		0.9974	0.9985	0.9999	0.9994	0.9995	0.9965	0.9997
Quality control standard concentrations (μM)	QC1	0.08	0.08	0.8	0.4	0.4	0.08	0.08
	QC2	0.25	0.25	2.5	1.25	1.25	0.25	0.25
	QC3	0.75	0.75	7.5	3.75	3.75	0.75	0.75
Accuracy (%)	Low	104	93.1	96	99.6	110	105	106
	Mid	112	105	108	114	108	115	107
	High	104	107.5	107	102	107	98.5	100
Precision (%)	Low	0.51	13.58	2.67	0.93	8.23	8.75	3.88
	Mid	2.27	5.39	2.30	4.51	6.39	5.47	4.55
	High	1.38	7.66	6.51	7.03	2.38	3.50	1.12
Recovery (%)	Low	109	92.4	92.3	101	105	111	103
	Mid	107	96.7	102	111	91.6	104	105
	High	96.2	90.2	91.7	100	97.6	104	102
Limit of detection (μM)		0.0006	0.0008	0.0330	0.0060	0.0010	0.0003	0.0007
Limit of quantitation (μM)		0.0020	0.0027	0.1100	0.0200	0.0033	0.0010	0.0023

Table S2: Average concentrations (µM) of water-soluble vitamins for reconstituted skim milk (9.5% total solids), sweet whey (6.5% total solids), micellar casein whey (6.5% total solids) and acid whey (6.5% total solids) powders derived from the milk of Holstein-Friesian cows assigned to perennial ryegrass (GRS), perennial ryegrass/white clover (CLV) and total mixed ration (TMR) feeding systems, determined by LC-MS/MS.

Sample type	Water-soluble vitamin (µM)	GRS	CLV	TMR
Skim milk powder	B1	0.72^{b}	0.72^{b}	0.55^{a}
	B2	39.4^{b}	40.5^{b}	22.6^{a}
	B3	0.24	0.18	0.26
	B3-amide	4.42	4.44	5.00
	B5	18.0	18.9	19.3
	B6-Pyridoxine	0.03	0.02	0.03
	B7	0.07	0.08	0.03
Sweet whey powder	B1	0.65	0.63	0.83
	B2	23.2^{b}	22.1^{b}	10.2^{a}
	B3	0.22^{a}	0.16^{a}	0.27^{b}
	B3-amide	3.60	3.99	4.06
	B5	19.8	18.8	18.7
	B6-Pyridoxine	0.02	0.03	0.03
	B7	0.07^{a,b}	0.08^{b}	0.03^{a}
Micellar casein whey powder	B1	0.45	0.36	0.41
	B2	2.60^{b}	2.71^{b}	1.17^{a}
	B3	0.19	0.16	0.21
	B3-amide	4.88	5.40	5.80
	B5	20.3	21.4	19.1
	B6-Pyridoxine	0.02	0.02	0.02
	B7	0.08^{b}	0.10^{b}	0.04^{a}
Acid whey powder	B1	0.56	0.43	0.52
	B2	1.05	1.13	0.62
	B3	0.23	0.17	0.31
	B3-amide	4.37^{a}	4.78^{a,b}	4.98^{b}
	B5	20.5	19.4	20.1
	B6-Pyridoxine	0.03	0.02	0.02
	B7	0.07^{b}	0.08^{b}	0.04^{a}

Values are presented as the mean of duplicate samples. GRS – Cows fed perennial ryegrass only. CLV – Cows fed perennial ryegrass / white clover. TMR – Cows fed total mixed ration *ad-libitum*. Vitamins: B1 – Thiamine, B2 – Riboflavin, B3 – Nicotinic acid, B3-amide – Nicotinamide, B5 – Pantothenic acid, B7 – Biotin. Note: Only the pyridoxine form of vitamin B6 is represented in the data. ^{a,b} different superscripts within a row indicate significant differences ($P<0.05$).

Table S3: Average concentrations (μM) of total water-soluble vitamins for reconstituted sweet whey (6.5% total solids), micellar casein whey (6.5% total solids) and acid whey (6.5% total solids) powders derived from the milk of Holstein-Friesian cows assigned to each feeding system, determined by LC-MS/MS.

Water-soluble vitamin (μM)	Sweet whey powder	Micellar casein whey powder	Acid whey powder
B1	0.70[b]	0.41[a]	0.50[a]
B2	18.5[b]	2.16[a]	0.93[a]
B3	0.22	0.18	0.23
B3-amide	3.88[a]	5.36[b]	4.71[b]
B5	19.1	20.2	20.0
B6-Pyridoxine	0.03	0.02	0.03
B7	0.06	0.07	0.06

Values are presented as the mean of duplicate samples. Vitamins: B1 – Thiamine, B2 – Riboflavin, B3 – Nicotinic acid, B3-amide – Nicotinamide, B5 – Pantothenic acid, B7 – Biotin. Note: Only the pyridoxine form of vitamin B6 is represented in the data. [a,b] different superscripts within a row indicate significant differences (P<0.05).

Table S4. Average concentrations (µg/g protein) of water-soluble vitamins for sweet whey, micellar casein whey and acid whey powders derived from the milk of Holstein-Friesian cows assigned to from perennial ryegrass (GRS), perennial ryegrass/white clover (CLV) and total mixed ration (TMR) feeding systems, determined by LC-MS/MS.

Diet	Ingredient type	Water-soluble vitamin (µg/g protein)						
		**B1**	**B2**	**B3**	**B3-amide**	**B5**	**B6-Pyridoxine**	**B7**
GRS	Sweet whey powder	$29.5^{a,b,c,d}$	1489^{c}	4.60	74.9^{a}	738	0.71	$3.04^{a,b,c}$
	Micellar casein whey powder	$23.2^{a,b,c}$	$191^{a,b}$	4.48	$117^{a,b,c}$	869	0.77	3.97^{c}
	Acid whey powder	$30.9^{a,b,c,d}$	81.7^{a}	5.89	$110^{a,b,c}$	929	1.03	$3.55^{a,b,c}$
CLV	Sweet whey powder	$27.9^{a,b,c,d}$	1400^{c}	3.37	$82.0^{a,b}$	695	0.77	$3.12^{b,c}$
	Micellar casein whey powder	22.1^{a}	$232^{a,b}$	4.51	$150^{b,c}$	1067	0.86	5.65^{c}
	Acid whey powder	$22.4^{a,b}$	83.8^{a}	4.03	$115^{a,b,c}$	843	0.77	$3.75^{b,c}$
TMR	Sweet whey powder	36.3^{d}	636^{b}	5.41	$81.9^{a,b}$	675	0.77	1.23^{a}
	Micellar casein whey powder	20.9^{a}	85.2^{a}	4.91	137^{c}	811	0.74	$1.77^{a,b}$
	Acid whey powder	$29.4^{a,b,c}$	49.6^{a}	8.03	$129^{a,b,c}$	933	0.80	$1.95^{a,b}$

Values are presented as the average of duplicate samples. GRS – Cows fed perennial ryegrass only. CLV – Cows fed perennial ryegrass / white clover. TMR – Cows fed total mixed ration *ad-libitum*. Vitamins: B1 – Thiamine, B2 – Riboflavin, B3 – Nicotinic acid, B3-amide – Nicotinamide, B5 – Pantothenic acid, B7 – Biotin. Note: Only the pyridoxine form of vitamin B6 is represented in the data. ^{a,b,c,d} different superscripts within a column indicate significant differences ($P<0.05$).
